# Response of Rice Genotypes to Weed Competition in Dry Direct-Seeded Rice in India

**DOI:** 10.1155/2014/641589

**Published:** 2014-06-30

**Authors:** Gulshan Mahajan, Mugalodi S. Ramesha, Bhagirath S. Chauhan

**Affiliations:** ^1^Punjab Agricultural University, Ludhiana 141004, India; ^2^International Rice Research Institute, India Office, Hyderabad 500030, India; ^3^Queensland Alliance for Agriculture and Food Innovation (QAAFI), University of Queensland, Toowoomba, QLD 4350, Australia

## Abstract

The differential weed-competitive abilities of eight rice genotypes and the traits that may confer such attributes were investigated under partial weedy and weed-free conditions in naturally occurring weed flora in dry direct-seeded rice during the rainy seasons of 2011 and 2012 at Ludhiana, Punjab, India. The results showed genotypic differences in competitiveness against weeds. In weed-free plots, grain yield varied from 6.6 to 8.9 t ha^−1^ across different genotypes; it was lowest for PR-115 and highest for the hybrid H-97158. In partial weedy plots, grain yield and weed biomass at flowering varied from 3.6 to 6.7 t ha^−1^ and from 174 to 419 g m^−2^, respectively. In partial weedy plots, grain yield was lowest for PR-115 and highest for PR-120. Average yield loss due to weed competition ranged from 21 to 46% in different rice genotypes. The study showed that early canopy closure, high leaf area index at early stage, and high root biomass and volume correlated positively with competitiveness. This study suggests that some traits (root biomass, leaf area index, and shoot biomass at the early stage) could play an important role in conferring weed competitiveness and these traits can be explored for dry-seeded rice.

## 1. Introduction

Dry-seeded rice (DSR) is an emerging rice production system in the northwestern Indo-Gangetic Plains amidst a looming water crisis and labor scarcity [1]. Weeds are among the most important biological constraints to successful production of DSR and, therefore, the cultivation of DSR warrants intensive use of herbicides for weed control [2]. DSR production systems are subject to greater weed pressure than conventional production systems, in which weeds are suppressed by flooding and transplanted rice seedlings have a “head start” over germinating weed seedlings. Many options exist for weed control in DSR, perhaps the most common being the use of herbicides. Reducing farmers' dependence on herbicides is desirable to reduce herbicide costs, minimize environmental pollution, and delay the evolution of herbicide-resistant weeds [1, 3]. In DSR, effective weed control requires proper herbicide application timing and method [4], which are often not met, resulting in poor weed control. Alternative weed management technologies are therefore much needed [5].

In herbicide-dominant systems, overall weed control efficiency can be improved when herbicides were combined with crop species or genotypes of superior competitiveness [6, 7]. Variation among genotypes in their ability to compete with weeds has been documented for many crops, including rice [8, 9]. Although some studies exist on the differences in competitiveness, including attempts on deciphering rice traits related to weed competitiveness and yield [10–12], only a limited number of cultivars have been evaluated so far.

Crop competitiveness against weeds is composed of tolerance to weed infestation, which is the ability to maintain high yields under weedy conditions, and weed-suppressive ability, which is the capacity to suppress weed growth in terms of dry matter [13]. Screening weed-competitive genotypes could offer an opportunity for using them as a component of integrated weed management strategies in DSR. However, to date, only few genotypes of rice for instance Haefele et al. [14] with superior weed competitiveness are known. Hybrids, due to their early vigor, may have the potential to complement the limited set of available competitive germplasm for DSR. The most important breeding objectives for the DSR genotypes were yield potential, high environmental adaptation, early vigor, and potentially favorable growth traits for weed suppression. Empirical evidence of superior performance of hybrids and new inbred lines adapted to DSR, in particular, the ability to better cope with weeds, is still awaited. Various authors suggested the evaluation of hybrids in DSR to confirm possession of weed-competitive traits and provide farmers with a wider choice of options when cultivating DSR [15].

Morphological, physiological, and biochemical traits are thought to control plant competitiveness [16] and many studies have been conducted to determine plant characters conferring competitive ability in cereals. Plant height plays a role in the competitive ability of rice [17]. Crop height appeared to have the greatest impact on competitive ability, with the shortest cultivars experiencing the largest yield reductions and allowing the greatest weed growth. However, height alone does not explain competitive ability because some shorter cultivars have been found to be good competitors in rice [18]. Some workers found that leaf area index (LAI) to be negatively correlated with specific leaf area, dry matter partitioning of leaves, and mean tip elevation angle [19]. They concluded that specific leaf area and tillering ability are major determinants of vegetative vigor. They further emphasized that vegetative vigor and crop duration, affecting the ability of genotypes to recover from early competition, are the useful traits in the selection of weed-competitive rice. Evidence that early season ground cover also reduces subsequent weed biomass has been reported by many researchers [20–22]. A better understanding of the mechanisms by which a rice genotype becomes more competitive to weeds would not only serve to assist plant breeders in developing competitive cultivars more quickly and effectively but would also justify the use of plant breeding to increase crop-competitive ability [8]. Yield gains of 7–9% have been identified in “competitive” aerobic cultivars when compared with “noncompetitive” cultivars [23].

The first objective of this study was to evaluate the weed competitiveness and yield potential of some popular hybrids and inbreds to identify superior genotypes that are potentially suitable for use in integrated weed management strategies in DSR. Second objective was to determine rice traits measured in weed-free and weedy conditions could be related to weed dry matter recorded under weedy conditions. This is the first study presenting a comprehensive assessment of weed-competitive ability of the popular rice genotypes/hybrids of this region, for the DSR system.

## 2. Materials and Methods

### 2.1. Site Description

Field studies were conducted to assess the effect of weed competition on the performance of rice genotypes in direct-seeded conditions in the rainy seasons (June–October) of 2011 and 2012 at the research farm of the Punjab Agricultural University, Ludhiana (30°56′N, 75°52′E), India. The climate of the region is semiarid, with an average annual rainfall of 400–700 mm (75–80% of which is received from July to September), a minimum temperature of 0–4°C in January and a maximum temperature of 41–45°C in June. The soil type at the experimental site was sandy loam with 0.3% organic matter and a pH of 7.2. The total N content in the soil was 0.042%. The available P (18 kg ha^−1^) and K contents (290 kg ha^−1^) in soil were medium and high, respectively. Field had the history of dry direct-seeded rice for 3 years. The weeds present in the field were* Commelina benghalensis *L.,* Cyperus rotundus *L.,* Dactyloctenium aegyptium *(L.) Willd.,* Digera arvensis *Forsk.,* Digitaria sanguinalis *(L.) Scop,* Echinochloa colona *(L.) Link,* Eragrostis *spp., and* Leptochloa chinensis *(L.) Nees. Weed composition was near to uniform in the entire field.

### 2.2. Experimental Design

The experiment in each year was established in a split-plot design with three replicates. The main plots included two levels of weed infestation (weed-free and partial weedy) and subplots (6.2 m × 1.4 m) included eight rice genotypes. Among genotypes, there were five inbred lines (two cultivars, PR-115 and PR-120; three advanced breeding lines, CR2707, IR83927, and IR88633) and three hybrids (H-97158, RH-257, and US-310). These genotypes were selected on the basis of yield in our previous breeding trials on DSR. All the selected genotypes were of* indica* rice.

In the weed-free plots, pendimethalin (750 g ai ha^−1^) as preemergence (PRE) was applied at 3 d after sowing (DAS) and bispyribac-sodium (25 g ai ha^−1^) as postemergence (POST) at 18 DAS. Following bispyribac-sodium application, plots were hand-weeded as needed to remove all weeds in the remainder of the season. Herbicides were applied using a knapsack sprayer with a flat fan nozzle and water as carrier at 375 L ha^−1^. In completely weedy plots, yield losses in DSR are greater than 90% [24]. In addition, it is not common for farmers to leave their rice fields infested with weeds in irrigated areas. The middle of the critical weed-free period in DSR is around 28 DAS [24]. Therefore, to make partial weedy plots, the weedy plots were hand-weeded once at 28 DAS and weeds were allowed to grow before and after the hand-weeding throughout the season [8].

### 2.3. Crop Management

Fields were prepared by cultivating twice using a disc harrow, followed by leveling with a wooden board. Seeds were sown by a single-row drill at a seeding rate of 30 kg ha^−1^ at 20-cm row spacing on June 21, 2011 and June 16, 2012. The field was surface-irrigated immediately after sowing. Nitrogen (N) fertilizer at 130 kg ha^−1^ (as urea) was applied in four equal splits at 14, 28, 49, and 70 DAS. Recommended rates of chlorpyrifos (500 g ai ha^−1^) and propiconazole (62.5 g ai ha^−1^) were used to control insect pests and diseases, respectively.

The LAI at tillering, panicle initiation, and flowering was measured with a digital plant canopy imager (model CI/110/CI-120, CID, Inc., Camas, WA), but the data are shown only for the tillering stage (28 DAS) due to similar trend. Under partial weedy condition, digital plant canopy imager also assesses the LAI of crop and weeds, so LAI was only recorded under weed free conditions. Measurements were made with the probe set parallel to the rows at two fixed locations in each plot. Two plants were randomly selected from each plot at the early ripening stage for root sampling. Root samples were collected by removing soil to a depth of 45 cm, along with the plants, with a 10-cm-diameter auger. A uniform soil volume (3,534 cm^3^) was excavated to collect root samples from all the treatments. Roots were carefully washed and various parameters (root biomass and volume) were measured and calculated. Root volume was measured using the water displacement method.

Two quadrats, 0.25 m² size, were placed at random in each plot to determine weed biomass at 28 DAS and at flowering. Only at 28 DAS, weeds were counted species-wise and differentiated into categories of sedges, grass, and broadleaf weeds. Weeds were cut at ground level, washed with tap water, sun-dried and then oven-dried at 70°C until constant weight, and weighed. For crop biomass, plant samples were collected in quadrats (0.40 m × 0.25 m) placed randomly at two locations in each plot at tillering, panicle initiation, and flowering stages. The plants were cut at ground level, oven-dried at 70°C for 72 h, and weighed. Grain yield was measured at 14% moisture from a sampling area of 5.2 m^2^ per plot. At the same time, five plants were selected randomly from each plot to measure agronomic parameters, which included plant height, grains panicle^−1^, and spikelet sterility (%). Competitiveness was measured as weed competitive index (CI) and calculated as [25, 26]

(1) $$\begin{array}{c} \text{CI}=\frac{\left[V_{\text{infest}}/V_{\text{mean}}\right]}{\left[W_{i}/W_{\text{mean}}\right]}, \end{array}$$

where *V*_infest_: is yield of variety (*i*) in terms of weed infested. *V*_mean_: is the average yield of all varieties in the presence of weed. *W*_*i*_: is weed biomass varieties of *i*. *W*_mean_: is average weed biomass is mixed with all varieties.

Crop vigor, recorded as visually rated crop biomass at 2 week after sowing on a per-plot basis on a 1-to-9 scale, where 9 was the greatest crop biomass and 1 was the least [8]. The relative yield loss (YL) of the crop challenged by weed competition under field conditions was estimated using equation, YL (%) = 1 − (*Y*_CW_/*Y*_CM_) × 100, where *Y*_CW_ and *Y*_CM_ are crop yields in competition with weeds and in weed-free conditions, respectively.

### 2.4. Statistical Analyses

In a combined analysis of data, the interactions of years with the level of weed infestation and/or genotypes were nonsignificant; therefore, the data were pooled over the years (therefore, a total of six replications) for further analyses (GenStat 8.0). Treatment means were separated using the least significant difference (LSD) test at the 5% level of significance. The relationships between grain yield (t ha^−1^) and various crop traits/parameters were assessed using linear regression analysis (SigmaPlot 10.0).

## 3. Results and Discussion

### 3.1. Weed Composition

Ten weed species were frequently observed in the weedy plots during both years. The most dominant weed species (on the basis of density) encountered in the weedy plots at 28 DAS in both years were* Cyperus iria *L. (21%),* Cyperus rotundus *(7%),* Echinochloa colona *(10%),* Alternanthera sessilis *(L.) R. Br. ex DC (4%),* Leptochloa chinensis *(12%),* Digitaria sanguinalis *(21%),* Dactyloctenium aegyptium *(6%),* Digera arvensis* (5%),* Commelina benghalensis *(5%), and* Eragrostis *spp. (9%). Genotype CR-2707 had the highest sedges, grasses, and broad-leaf density among all the tested genotypes (Table 1). Sedges, grasses, and broadleaf weed density were similar for genotypes PR-120, IR88633, and RH-257, however lower than CR-2707 and H-97158. Genotypes PR-115 and IR-83927 had similar sedges, grasses, and broadleaf weed density but lower than that of CR-2707. Weed species found in the trial do prefer upland rather than flooded conditions. The field was exposed to alternate wetting and drying conditions and these conditions are likely to favor species diversification depending upon the competitive ability of the genotypes. In DSR, weed composition is generally found to be more diverse than in transplanted rice [4].

### 3.2. Weed Biomass and Grain Yield

At 28 DAS, H-97158, and CR2707 accrued with higher weed biomass than other genotypes (Table 2). At this stage, lowest weed biomass was recorded in PR-120. Weed biomass at flowering was also lowest in PR-120 and it was highest in hybrid H-97158 (Table 2). Genotypes IR88633, IR83927, PR-120, and RH-257 accrued with similar but lower weed biomass than that of H-97158. Genotypes US-310, CR2707, and H-97158 accrued with similar weed biomass; however, they had higher weed biomass than PR-120. PR-115 had weed biomass similar to that of IR88633, IR83927, and RH-257.

Weed competition caused significant reduction in grain yield of all the tested genotypes and the reduction was positively correlated with weed biomass (*r* = 0.65; *P* = 0.40). Grain yield in weedy plots varied from 3.6 to 6.7 t ha^−1^ across different genotypes and it was lowest and highest for PR-115 and PR-120, respectively. However, in weed-free plots, grain yield varied from 6.6 to 8.9 t ha^−1^ across different genotypes and it was lowest for PR-115 and highest for H-97158 (Table 3). Average yield loss due to weed competition ranged from 21 to 46% among different genotypes. The higher the weed biomass, the greater the percent yield reduction. The highest relative yield loss due to weed competition was noted in PR-115 and the relative yield loss of this cultivar was similar to that of H-97158, CR2707, US-310, and RH-257. The lowest relative yield loss due to weed competition was in PR-120, which was similar to that of IR88633 and IR83927. These results suggest that rice genotypes responded differently in their competitiveness in suppressing weeds under severe infestation. PR-120, IR88633, and IR83927 proved to be better weed competitors than the other genotypes.

Correlation analyses were used to explore the relationship between grain yield of different rice genotypes and plant characteristics in both weed-free and partial weedy conditions. In weed-free conditions, only grain yield had a significant positive correlation with panicle density m^−2^ (*r* = 0.77) (Table 4). In partial weedy conditions, however, grain yield had a significant positive correlation with panicle density m^−2^, grain panicle^−1^, weed competitive index, LAI, root weight, and root volume (Table 5). These results indicate that these parameters could have played a vital role in improving grain yield under crop-weed competition. In partial weedy conditions, grain yield was negatively related with spikelet sterility, relative yield loss, and weed biomass.

**Figure 1:**
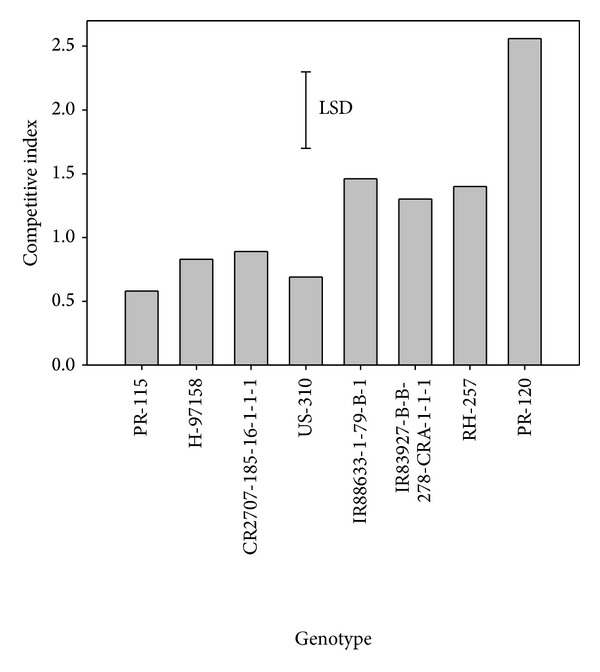
Weed competitive index of different rice genotypes.

In weed-free plots, the highest grain yield was found in H-97158 followed by RH-257. This was attributed to higher panicle density in H-97158. Lowest grain yield was recorded in PR-115. Weed competitive index was highest for PR-120 and other genotypes had lower weed competitive index than this cultivar (Figure 1). PR-115 had the lowest weed competitive index, which was similar to that of genotypes H-97158, CR 2707, and US-310. On the other hand, IR88633, IR83927, and RH-257 had better weed competitive index than PR-115. The high performance of these genotypes in terms of weed competitive index could be attributed to less weed biomass observed due to their ability to suppress weeds. Weeds can be suppressed by allelopathic effects of rice genotypes in addition to genotypic smothering effect on weeds; however, the genotypes used in our study have not been reported for their allelopathic effect.

The interaction effects of weed infestation levels and genotypes were significant for grain yield, panicle number m^−2^, and grain number panicle^−1^ (Table 3). Across weed infestation levels, 1000-grain weight was highest for IR88633 followed by IR83927 (Table 6). Averaged over weed infestation levels, 1000-grain weight of all the other genotypes was similar to but lower than that of IR88633. In the partial weedy conditions, 1000-grain weight had a positive correlation with weed competitive index, whereas such correlation was not found in weed-free conditions. Plant height differed across the genotypes (Table 6). IR88633 attained the highest plant height followed by IR83927 and CR2707. Lowest plant height was found for PR-115. Genotypes H-97158, US-310, and RH-257 attained similar plant height. Under partial weedy conditions, plant height had a negative correlation with relative yield loss.

As expected, grain yield of all genotypes in weedy conditions decreased significantly as compared with yield in weed-free conditions (Table 3). In partial weedy conditions, highest grain yield was found for PR-120, closely followed by IR83927. Grain yield of PR-120 in partial weedy conditions was similar to that of PR-115, US-310, and IR88633 in weed-free conditions. This response was because of the better competitive ability of PR-120; that is, it had the highest weed competitive index (Figure 1) and root biomass and volume (Table 7). Root biomass and volume were highest in PR-120 and lowest in PR-115; however, these root parameters of PR-115, H-97158, CR2707, and US-310 were the same. Similarly, IR88633, IR83927, and RH-257 had almost identical root biomass and volume. High root biomass and volume offered crop-weed competition in favor of the crop, providing greater vigor and early canopy closure as evidenced by the higher LAI of these genotypes.

Vigor score was highest in cultivars IR88633 and CR2707 (Table 7). Among the tested genotypes, vigor score was lowest for PR-115, followed by H-97158 and US-310. Early vigor has been shown as an important characteristics related to competitive ability [16] and tends to be reflected in greater shoot and root biomass during early vegetative growth. In the present study, crop biomass of PR-120 remained similar in both partial weedy and weed-free conditions (Table 8). At panicle initiation, PR-120 in the partial weedy conditions accrued with similar crop biomass with H-97158 and IR88633 in the weed-free conditions. In the partial weedy conditions, at panicle initiation and flowering stages, IR88633, IR83927, RH-257, and PR-120 accrued with higher crop biomass than that of PR-115, H-97158, CR2707, and US-310, respectively (Table 8).

The present study revealed that higher biomass, coupled with higher plant height at early stages, provided greater stored assimilates for grain filling via translocation that may prevent the yield loss of these genotypes under partial weedy conditions. In partial weedy conditions, grain number panicle^−1^ remained similar only in IR83927 and PR-120 as compared with that in weed-free conditions, and this response was mainly because of their better weed-competitive ability. In partial weedy conditions, the highest number of grains panicle^−1^ was found in PR-120. Grain number panicle^−1^ for PR-120 in partial weedy conditions was similar to that of PR-115, H-97158, IR88633, and IR83927 in the weed-free conditions.

High vigor score and LAI during early crop development have been shown to be major plant traits contributing to weed competitiveness [27, 28]. The LAI is an important contributor to competitive ability, as light is an important resource in plant growth. Greater shading ability of the crop makes less light available to the competing weeds; hence, weed growth can be reduced. In the weed-free plots, IR88633 attained the highest LAI at 28 DAS, whereas PR-115 attained the lowest LAI (Table 7). The LAI of PR-115 and US-310 was similar. Genotypes H-97158, RH-257, PR-120, and IR83927 recorded similar LAI, but this was higher than that of PR-115. The LAI of CR2707 and IR88633 was similar.

The findings of our study indicate that fast-growing habit and the high LAI at the early stage of the crop, along with high root biomass and volume, are the important traits for weed competitiveness, confirming studies by various authors [18, 29]. However, some workers [30] demonstrated that an advantage of early growth habit, even for a few days, could shift the competitive balance between crops and weeds. In another study, some workers found a significant correlation between crop competitive ability and plant height [31]. In the present study, a negative correlation was found between plant height and relative yield loss. In contrast, Fischer et al. found no correlation between the height of semidwarf cultivars and weed biomass [11]. As in our study, research by some scientists [32] suggested that rice cultivars capable of producing more grain yield in competition with weeds have greater early biomass. Similarly, workers [8, 33] proposed that early vigor should be a key selection trait for weed competitiveness. PR-120, owing to its high root biomass and volume, which helped in attaining high crop biomass and greater panicle number m^−2^, produced the highest yield in partial weedy conditions. Our results confirm the findings of researchers, who reported significant and negative correlation between weed biomass and panicle number m^−2^, suggesting that panicle density was decreased by high weed growth [31].

Competitive ability is the ability of a genotype to suppress weed biomass or to maintain crop yields by tolerating weed competition. In this study, competitive ability was found highest for PR-120. The competitiveness of a cultivar might be due to the higher number of panicles m^−2^, early growth, and yield under both partial weedy and weed-free conditions. In this study, panicle number m^−2^ also had a correlation with grain yield under both partial weedy and weed-free conditions. Cultivars able to produce higher grain yields in partial weedy conditions correlated with greater early rice growth and LAI [8, 34, 35]. Likewise, this study demonstrated that high LAI, along with high root biomass and volume, were the useful traits for weed-competitive genotypes. CR-2707 and RH-257 had higher LAI and vigor, and similarly, RH-257 had higher root biomass and volume, but yield loss of these genotypes was found more than 35%. This was due to their (CR-2707 and RH-257) more tendencies for lodging under partial weedy conditions.

In summary, genotypes PR-115 and H-97158 were the worst competitors and PR-120, IR88633, and IR83927 were good weed competitors. Genotypes PR-120, IR88633, and IR83927 have potential for increasing the weed-competitive ability of genotypes in breeding programs and could be used to increase the competitiveness of highly productive genotypes that are not competitive. The study revealed that early canopy closure, high LAI at the early stage, and greater root biomass and volume correlated positively with competitiveness. This study demonstrated that breeding to increase the competiveness of highly productive rice plant types would be possible without compromising yield. The competitiveness observed in these studies for genotypes PR-120, IR88633, and IR83927 would be adequate in improving farmers' income and reducing herbicide use and can be successfully used in integrated weed management programs in DSR.

## 4. Future Implications

This study implies that breeding for weed-suppressive rice genotypes should be conducted under partial weedy conditions. In a mixed weed population, the weed-competitive ability of rice genotypes may be affected by weed composition. However, the competitive ability of rice genotypes is a complex trait and could not be explained by only one or two characteristics. Interactions between traits, for instance, root biomass, LAI, and high shoot biomass at early stage, play an important role and these traits can be explored for identifying weed-competitive genotypes in DSR. Competitiveness of rice genotypes against weeds will be an important key to the successful adoption of weed-suppressive rice genotypes in a sustainable weed management program. The development of such genotypes could play an important role in DSR by reducing herbicide load in agroeco system.

## Figures and Tables

**Table 1:** Weed density (number m^−2^) in response to different genotypes at 28 days after sowing.

Genotype	Weed density		
	Grasses	Broadleaved	Sedges
	number m^−2^		
PR-115	42	20	24
H-97158	54	25	31
CR2707	61	29	35
US-310	36	17	20
IR88633	26	12	15
IR83927	39	18	22
RH-257	32	15	18
PR-120	30	14	17
LSD (0.05)	7	3	4

**Table 2:** Weed biomass (g m^−2^) in response to different genotypes at 28 d after sowing (DAS) and flowering.

Genotype	Weed biomass	
	28 DAS	Flowering
	g m^−2^	
PR-115	85.4	333.0
H-97158	117.5	418.5
CR2707	125.7	386.5
US-310	79.4	381.2
IR88633	56.7	221.1
IR83927	70.6	280.8
RH-257	61.3	238.0
PR-120	44.8	174.3
LSD (0.05)	26.1	118.4

**Table 3:** Grain yield (t ha^−1^), panicles m^−2^ and grains panicle^−1^ in response to the interaction effect of weed infestation levels and genotypes.

Genotype	Grain yield		Panicles		Filled grains	
	Weed-free^a^	Partial weedy	Weed-free	Partial weedy	Weed-free	Partial weedy
	t ha^−1^		number m^−2^		number panicle^−1^	
PR-115	6.59	3.58	290	230	114	96
H-97158	8.89	5.15	351	236	133	103
CR2707	7.43	4.80	305	238	136	96
US-310	6.78	4.52	252	247	148	95
IR88633	6.95	5.20	278	249	130	91
IR83927	8.09	6.03	336	259	122	108
RH-257	8.63	5.50	340	252	136	115
PR-120	8.47	6.66	362	308	124	120
LSD (0.05)	0.7		29		14	

^a^Weed-free: plots were kept weed-free throughout the season. Partial weedy plots: plots were hand-weeded once at 28 DAS and weeds were allowed to grow before and after the hand-weeding throughout the season.

**Table 4:** Correlation among different traits in weed-free conditions. The critical value of *r* was 0.40 at 5% level of significance.

Traits	GY	Pan	Gr	TGW	SS	Ht	CI	RYL	WP	LAI	VS	RW
GY												
Pan	0.77											
Gr	−0.06	−0.30										
TGW	0.13	−0.03	−0.16									
SS	−0.36	−0.41	−0.09	−0.39								
Ht	−0.05	−0.24	0.11	0.77	−0.28							
CI	0.25	0.35	−0.01	0.20	−0.56	0.09						
RYL	−0.08	−0.21	−0.02	−0.41	0.44	−0.22	−0.69					
WP	−0.02	−0.19	0.28	−0.33	0.32	−0.13	−0.78	0.65				
LAI	0.39	0.32	0.05	0.62	−0.66	0.62	0.60	−0.39	−0.40			
VS	0.01	−0.04	−0.09	0.60	−0.38	0.59	0.35	−0.30	−0.17	0.72		
RW	0.32	0.45	−0.15	0.41	−0.49	0.10	0.58	−0.64	−0.56	0.48	0.37	
RV	0.32	0.45	−0.15	0.41	−0.49	0.10	0.58	−0.64	−0.56	0.48	0.37	1.00

GY, grain yield; LAI, leaf area index at 28 DAS; Ht, height; RW, root weight; RV, root volume; Pan, panicle m^−2^; Gr, filled grains panicle^−1^; TGW, 1000-grain weight; SS, spikelet sterility; CI, weed competitive index; WP; weed biomass; and RYL, relative yield loss.

**Table 5:** Correlation among different traits in partial weedy conditions. The critical value of *r* was 0.40 at 5% level of significance.

Traits	GY	Pan	Gr	TGW	SS	Ht	CI	RYL	WP	LAI	VS	RW
GY												
Pan	0.61											
Gr	0.53	0.62										
TGW	0.33	0.18	0.00									
SS	−0.47	−0.34	−0.39	−0.38								
Ht	0.23	0.01	−0.17	0.78	−0.24							
CI	0.71	0.32	0.26	0.21	−0.49	0.17						
RYL	−0.84	−0.51	−0.31	−0.41	0.26	−0.42	−0.69					
WP	−0.56	−0.29	−0.27	−0.34	0.32	−0.20	−0.78	0.65				
LAI	0.51	0.14	0.14	0.59	−0.62	0.63	0.60	−0.39	−0.40			
VS	0.22	0.08	−0.10	0.62	−0.41	0.70	0.35	−0.30	−0.17	0.72		
RW	0.70	0.63	0.38	0.55	−0.47	0.33	0.58	−0.64	−0.56	0.48	0.37	
RV	0.70	0.63	0.38	0.55	−0.47	0.33	0.58	−0.64	−0.56	0.48	0.37	

GY, grain yield; LAI, leaf area index at 28 DAS; Ht, height; RW, root weight; RV, root volume; Pan, panicle m^−2^; Gr, filled grains panicle^−1^; TGW, 1000-grain weight; SS, spikelet sterility; CI, weed competitive index; WP; weed biomass; and RYL, relative yield loss.

**Table 6:** Final plant height (cm plant^−1^), 1000-grain weight (g), and spikelet sterility (%) in response to weed infestation levels and genotypes.

Treatment	Plant height	1000-grain weight	Spikelet sterility
	cm	g	%
Weed infestation level			
Weed-free^a^	115.3	26.0	19.5
Partial weedy	116.5	25.6	20.1
LSD (0.05)	NS	NS	1.6
Genotype			
PR-115	93.5	24.7	26.2
H-97158	109.4	25.5	18.2
CR2707	124.6	25.4	15.7
US-310	109.2	24.3	25.8
IR88633	150.2	28.9	16.6
IR83927	126.9	27.0	17.1
RH-257	112.6	25.8	19.4
PR-120	100.1	25.5	14.6
LSD (0.05)	7.1	0.5	3.9

Nonsignificant, NS.

^a^Weed free plots: plots were kept weed free throughout the season. Partial weedy plots: plots were hand-weeded once at 28 DAS and weeds were allowed to grow before and after the hand-weeding throughout the season.

**Table 7:** Leaf area index (LAI) and vigor score at 28 days after sowing and root biomass and volume at flowering of various genotypes under weed-free conditions.

Genotype	LAI	Vigor score	Root biomass	Root volume
			g m^−2^	mL m^−2^
PR-115	1.86	4.3	147.8	561.5
H-97158	2.73	4.7	200.9	763.5
CR2707	3.26	9.0	196.5	746.6
US-310	1.90	4.7	175.7	667.6
IR88633	3.37	9.0	342.8	1302.7
IR83927	2.89	7.0	291.4	1107.3
RH-257	3.10	6.7	303.4	1153.0
PR-120	2.82	6.7	429.5	1653.8
LSD (0.05)	0.43	0.6	75.5	287.0

**Table 8:** Crop biomass (t ha^−1^) in response to the interaction effect of weed infestation levels and genotypes at panicle initiation and flowering stages.

Weed infestation level	Crop biomass							
	PR-115	H-97158	CR2707	US-310	IR88633	IR83927	RH-257	PR-120
	t ha^−1^							
Panicle initiation stage								
Weed-free^a^	4.3	4.8	3.20	3.80	4.90	4.40	4.50	4.60
Partial weedy	3.30	3.25	3.15	3.40	4.22	4.14	4.40	4.80
LSD (0.05)	0.3							

Flowering stage								
Weed-free	6.98	7.48	6.98	7.21	8.02	7.12	7.32	7.18
Partial weedy	3.82	4.25	4.10	4.12	4.90	5.12	5.30	5.92
LSD (0.05)	0.5							

^a^Weed free plots: plots were kept weed free throughout the season. Partial weedy plots: plots were hand-weeded once at 28 DAS and weeds were allowed to grow before and after the hand-weeding throughout the season.
